# The Tenth Annual Report of the Secretary of the State Board of Health of the State of Michigan

**Published:** 1883-05

**Authors:** 


**The Tenth Annual Report of the Secretary of the State Board of Health of the State of Michigan, for the fiscal year ending April 30, 1882.** By authority. Lansing: W. S. George & Co., State Printer and Binder. 1883.

The Peninsular State, in its efforts to advance the sanitary interests of its people, outstrips many of older communities in this very important department of scientific study and research. The present volume contains many very valuable papers on sanitary science, which show great labor and study as well as patient investigation. The volume contains the records and papers of two sanitary conventions, the one held at Ann Arbor, and the second at Greenville. Among the writers we find many of the ablest men in the State. Judge Cooley writes a very able paper on "What can the Law do for the Health of the People;" Prof. Lyster, of the State University, on the "Ambulance Hospital for Small Pox;" Rev. Dr. Duffield on "Hygiene and the Clerical Profession;" Prof. J. W. Langley on "Ventilation;" Prof Prescott, of the State University, on "Food Adulterations," and Prof. V. C. Vaughn, of the same institution, on "Meats." We notice a very interesting paper on "Health of Criminal Women," by Eliza M. Mosher, M. D., of the Sherborn Reformatory for women, which reveals some startling statistics in regard to the criminal class of the fair sex. We quote: "It is safe to say of the whole number committed during the four years, more than four-fifths were intempecate, and more than three-fourths were unchaste. The paper further states that "one woman out of every four committed to the prison was syphilitic." The report is a very valuable record of earnest work in sanitary science. The character of the papers and the high-standing and ability of the writers, show that in scientific circles in that State there is constantly held in view the relations of *science* to the well-being and happiness of man. We commend such an example to the attention of the profession in this and other States.

